# A Langendorff-heart discovery pipeline demonstrates cardiomyocyte targeting by extracellular vesicles functionalized with beta-blockers using click-chemistry

**DOI:** 10.1016/j.yjmcc.2025.05.007

**Published:** 2025-05-23

**Authors:** Kyung Chan Park, Amir Mashia Jaafari, Christopher Anthony Smith, Althea Rennisa Lobo, Lorenzo Errichelli, Gül Şimşek, Mala Gunadasa-Rohling, Alexander Marchant, Maria O. Levitin, Virginia Castilla-Llorente, Patrick Vilela, Pawel Swietach

**Affiliations:** a Department of Physiology, Anatomy & Genetics, Parks Road, Oxford OX1 3PT, UK; b Evox Therapeutics, Medawar Centre, Robert Robinson Ave, Oxford OX4 4HG, UK; c Department of Biophysics, Faculty of Medicine, Ankara University, Ankara, Türkiye

**Keywords:** Beta-receptors, Myocardium, Drug delivery, Langendorff perfusion, Screening

## Abstract

Extracellular vesicles (EVs) are widely explored as vehicles for delivering therapeutic or experimental cargo to cardiomyocytes. Efforts to improve EV bioavailability in the heart, and reduce their off-target actions, require screening methods that can replicate the physiological and anatomical barriers present in the myocardium. Additionally, discovery pipelines must exercise control over EV dosage and timing, and provide a means of assessing cargo incorporation into cardiomyocytes specifically. These criteria are not generally met by experiments on cultured cells or animals. Here, we present a Langendorff-heart discovery pipeline that combines the strengths of *in vivo* and *in vitro* approaches. Langendorff-mode perfusion enables controlled exposure of beating hearts to re-circulated EVs. Following perfusion, cardiomyocytes can be isolated enzymatically for analysis, such as imaging. We tested this discovery pipeline by functionalizing EVs with beta-blockers (atenolol, metoprolol) using click-chemistry and incorporating the fluorescent protein NeonGreen2 to track the fate of EV cargo. Fluorescence in cardiomyocytes, including their nuclear regions, increased after Langendorff-treatment with beta-blocker decorated EVs, but only if these contained NeonGreen2, implicating the fluorescent cargo as the source of signal. Superior binding efficacy of beta-blockers was confirmed by referencing to the substantially lower signals obtained using wild-type EVs or EVs presenting myomaker or myomixer proteins, motifs that modestly enrich cardiac EV uptake in mice. Our findings demonstrate successful cardiomyocyte targeting using EVs decorated with beta-receptor binders. We propose the Langendorff-perfused heart as an intermediate step - nested between *in vitro* characterisation and animal testing - in discovery pipelines for seeking improved cardiacspecific EV designs.

## 1 Introduction

Extracellular vesicles (EVs) hold considerable potential for delivering therapeutic cargo to diseased tissues, including cardiomyocytes affected by metabolic, signalling, electrophysiological, or contractile disorders [1,2]. Compared to cell-based treatments, EVs have *(i)* a better safety profile and *(ii)* less onerous storage and transport requirements [3]. An obstacle for cardiac therapeutics is targeting efficiency, which limits therapeutic cargo incorporation into cardiomyocytes *via* membrane fusion and endocytosis [4,5]. A key area of development is refining the EV surface for better cardiomyocyte targeting [2]. Notable EV engineering efforts have involved homing peptides, such as CSTSMLKAC [6–8], and chemically decorating EVs with cardiomyocyte-binding moieties [7,9,10]. The success of these modifications can only be assessed through screening protocols, which have primarily entailed *in vivo* animal experiments on the premise that these replicate the barriers to delivery in patients [2]. Aside from being low-throughput, the *in vivo* approach is associated with significant attrition in the EV-load reaching the heart [11–15], with the majority of injected EVs dissipating into the liver, lungs and kidneys [16]. The extent of this loss is a variable that can compromise the interpretation of efficacy studies; for example, it may not be possible to ascribe poor treatment outcomes to low EV bioavailability or a weak biological action in the target cells. In summary, screening efforts to discover improved EV designs for the heart could benefit from improved control of key parameters: the concentration and exposure time at the target organ.

Efforts to overcome the shortcomings of screens have taken two strategies for cardiac therapeutics. In the first approach, intracoronary and intramyocardial deliveries were tested to bypass the systemic circulation [17–19]. However, these interventions are poorly calibrated, likely to produce only transient EV exposure, and therefore introduce experimental noise. The second approach would expose primary or immortalized cardiac cells to EVs *in vitro* [20]. Whilst this method offers fine control over concentration and exposure time, it eliminates key barriers to delivery present in the beating myocardium, notably blood vessels and the stroma surrounding cardiomyocytes. It is therefore timely to design an efficient screening platform that would control the timing and dosage of EVs and maintain the integrity of the beating heart. Since the myocardium is composed of various cell types [21], the screening platform should also provide a means of quantifying cargo uptake specifically into cardiomyocytes. To that end, we implemented a protocol that controls EV concentration and exposure time by Langendorff (retrograde) perfusion of a beating heart *ex vivo* [22], followed by enzymic digestion that isolates cardiomyocytes for subsequent assays. We used this method to test cardiomyocyte targeting of EVs functionalised with binders to β_1_-receptors present abundantly in the myocardium [23,24] and loaded with NeonGreen2 to image cellular uptake. β_1_-selective antagonists (also known as beta-blockers) were selected because of their avid binding to the myocardium (albeit with some off-target binding to the renal juxtaglomerular apparatus) [24], and favourable safety profile evidenced by their widespread clinical use [25]. Based on our findings, we propose Langendorff perfusion as an intermediate step in the EV development pipeline, nested between *in vitro* characterisation using myocytes and *in vivo* testing in animals, reasoning that it enables well-controlled protocols whilst retaining organ-level physiological relevance. As a demonstrable outcome, we show evidence that decorating EVs with beta-blockers can deliver cargo to cardiomyocytes.

## 2 Methods

### 2.1 Cell culture and EV generation

EVs were produced from CAP suspension cells (CEVEC, Germany) according to a modification of a published protocol [26]. Briefly, CAP cells were grown at 37 °C and 5 % CO_2_, in multiple 1 L-flasks and transfected with various sets of constructs: NanoLuc alone, NanoLuc alongside one of 8 muscle targeting motifs, including myomaker (MYMK) and myomixer (MYMX), NeonGreen2 (mNG) alone, mNG with MYMK, or mNG with MYMX. Each transfection used 1 pg of DNA per cell using PEI at a ratio of 1:2 DNA:PEI in Freestyle 293 media (Thermo-Fisher). After 6 h, media were changed to PEM (ThermoFisher) and 72 h were allowed for the generation of EVs.

### 2.2 EV harvest, purification and characterisation

EVs were harvested by spinning the cell suspension at 400 g for 30 min and collecting the supernatant. The supernatant was purified using tangential flow filtration (TFF), followed by a round of AKTA purification, and a final concentration step using TFF. EV concentration was calculated using Nanoparticle Tracking Analysis (NTA) [27] and characterisation was performed by western blotting. EVs were stored at −80 °C until use.

### 2.3 Animal procedures

The use of animals was approved by the University of Oxford ethical review board. To obtain hearts for Langendorff perfusion, 8–10 week old male FVB/NCrl mice were terminally anesthetized *via* intraperitoneal sodium pentobarbital injection and heparin was co-administered to reduce the risk of clot formation. Cervical dislocation was performed to confirm death. Hearts were rapidly excised and canulated at the aorta for Langendorff retrograde perfusions. To study biodistribution of EVs *in vivo*, male and female B6;129-Gaatm1Rabn/J mice (Pompe disease model) were obtained from a certified supplier and housed in an AAALAC-accredited facility under controlled environmental conditions (temperature, humidity, and a 12:12 h light/dark cycle). Animals were acclimated for at least 5 days prior to study initiation. For each experimental condition, mice were randomly assigned to treatment groups. NanoLuc-labeled EVs were supplied as 500 μL frozen aliquots and stored at − 60 °C. Before administration, individual aliquots were thawed at room temperature, gently vortexed, and briefly centrifuged to collect the solution. To preserve EV stability, thawed preparations were used within 2 h; any unused portion was returned to storage. Conscious mice received a single intravenous bolus injection *via* the lateral tail vein at a dose volume of 5 mL/kg. Animals were monitored continuously for any adverse effects. At 3 h post-dosing, mice were anesthetized with an intraperitoneal injection of a ketamine/xylazine mixture (100–150 mg/kg ketamine and 10 mg/kg xylazine) and subsequently euthanized using a terminal (Schedule 1) procedure. Retro-orbital blood samples (250 μL per animal) were collected using a heparinized capillary following full anesthesia. Blood samples were immediately transferred to heparinized tubes and processed by centrifugation (an initial spin at 2200 *g* for 10 min at room temperature, followed by a second spin at 2200 g for 5 min) to isolate plasma, which was then aliquoted and stored at −60 °C. Immediately after blood collection, mice underwent perfusion *via* left ventricular cannulation with sterile saline (50 mL delivered by gravity over 5 min) to minimize blood-borne EV contamination in subsequent tissue samples. Tissues, including the liver, heart, skeletal muscles, spleen, and lungs, were rapidly excised, snap-frozen in liquid nitrogen, and maintained at −60 °C until further analysis.

### 2.4 Bioluminescence measurement

Frozen tissue samples were weighed and immediately homogenized in PBS containing 0.1 % Triton X-100 using a bead-based homogenizer. The volume of lysis buffer was adjusted proportionally (0.05 mL per 10 mg tissue) to ensure consistent extraction across tissue types. Homogenates, along with aliquots of plasma and any remaining test article, were assayed for NanoLuc luciferase activity using the Promega Nano-Glo Luciferase Assay System, following the manufacturer’s instructions. Luminescence data were normalized to the administered NanoLuc dose and to the tissue mass or total protein content to enable direct comparisons across treatment groups.

### 2.5 Decoration with β_1_-selective antagonists

Where indicated, the EV surface was decorated with metoprolol (Me) and atenolol (At) molecules. Functionalization was achieved by adapting existing described protocols [28,29]. Briefly, metoprolol or atenolol were dissolved at 1 M in 1× PBS. Both molecules were activated using a modified protocol of EDC/sulfo-NHS (ThermoFisher, UK). Unreacted reagents were removed from solution using the AKTA system. Purified sulfo-drugs were then incubated with EVs (wild-type or expressing NeonGreen2) at 200 mM for 1 h at 37 °C. This was followed by a final purification of samples using AKTA. Drug-decorated EVs were characterised using qEV separation columns and fluorescence in NeonGreen2-expressing EVs was monitored using SpectraMax at 520 nm to confirm the presence of beta-blockers on the EV surface.

### 2.6 Langendorff perfusion

The aorta was cannulated, the heart dissected out of the chest cavity and immediately connected to a Langendorff perfusion system driven by a peristaltic pump (2 ml/min). The base solution for Langendorff perfusion contained 130 mM NaCl, 5.6 mM KCl, 5 mM HEPES, 0.45 mM NaH_2_PO4, 10 mM glucose, 20 mM taurine, and 3.5 mM MgCl_2_; pH was adjusted to 7.4 at 37 °C using NaOH. Initially, the heart was perfused for at least 1 min in base solution supplemented with 5 U/mL heparin to clear vessels from blood. For experiments involving “on-circuit” EV treatment, Langendorff-perfusion was returned to Heparin-free solution and valves were switched to re-circulation mode. Into the 20 mL re-circulating solution, CaCl_2_ was added to 1 mM and EVs were added to the desired concentration. The health of the heart was monitored from its contractile activity. After a desired period of EV re-circulation (typically 1 h), the heart was perfused with EV-free perfusate for three minutes to wash-away any unbound particles. For some experiments, the heart was prepared for fixing and immunofluorescence. For other experiments, the heart was digested to liberate cells by perfusion with base solution containing digestion enzymes (2.5 mg/ml Liberase) and 1 mM CaCl_2_. The timing of digestion was determined empirically to produce the highest yield of cells, and typically 9 min. Next, the digested heart was removed from the canula, atria discarded, and remaining mass was re-suspended in solution containing 1 % BSA (to block enzyme activity) and 0.2 mM CaCl_2_. The ventricles were minced using fine scissors and the supernatant containing the cardiomyocytes was filtered through a 450-μm filter into a Falcon tube. The residual mass was re-suspended in enzyme solution, warmed in a water bath at 37 °C for 5 min; the supernatant was filtered into a separate tube, and the process was repeated twice for remaining heart tissue. All myocyte suspensions were centrifuged and supernatants replaced with HEPES-buffered Tyrode containing 135 mM NaCl, 20 mM HEPES, 4.5 mM KCl, 1 mM MgCl_2_, 11 mM glucose and 1 mM CaCl_2_; pH was adjusted to 7.4 at 25 °C using NaOH. In some experiments, EVs were reintroduced to the suspension to continue the loading process and test for any added effect.

### 2.7 Immunofluorescence

Hearts were sectioned using a cryostat and sections were incubated with mouse anti-NeonGreen2 antibodies at concentrations 1:50, 1:100, 1:500, 1:1000 (ChromoTek, #32f6) and rabbit anti-α-actinin antibodies at 1:200 (Proteintech, #11313–2-AP). The secondary antibodies used were goat anti-mouse IgG Alexa Fluor 555 (Invitrogen, #A32727) and goat anti-rabbit IgG Alexa Flour 488 (Invitrogen, #A32731), both diluted to 1:200. Sections were also stained with DAPI. Sections were imaged on a Zeiss LSM 700 confocal microscope using excitation wavelengths 405, 488 and 555 nm to visualise DAPI, alpha-actinin and neon green respectively.

### 2.8 Imaging isolated myocytes

Cardiomyocytes were loaded with Hoechst (3.3 μM, 5 min) and aliquoted in a superfusion chamber with a coverslip glass bottom pretreated with poly-L-lysine. After allowing for cell adhesion, myocytes were superfused with calcium-free solution (to block contraction) at a rate of 4 ml/min and imaged on two channels using a Zeiss LSM700 confocal imaging system. This superfusate contained 135 mM NaCl, 20 mM HEPES, 4.5 mM KCl, 1 mM MgCl_2_, 11 mM glucose and 0.5 mM EGTA; pH was adjusted to 7.4 at 25 °C using NaOH. The first recording channel (405 nm excitation/480 nm emission) captured Hoechst staining of nuclei plus weak autofluorescence that is sufficient to demarcate the cell outline relative to the non-fluorescent background. The second recording channel (488 nm excitation/520 nm emission) was optimised for detecting NeonGreen2 fluorescence. Imaging was performed sequentially to eliminate bleed-through and at constant equipment settings (laser power, PMT gain, pinhole) to enable comparisons between different experiments.

### 2.9 Image analysis

Immunofluorescence images were analysed by masking the DAPI channel for nuclei, and the actinin channel for cells. Signal in the red channel was segmented by extracellular, cytoplasmic (cells minus nuclei), and nuclear. Images of isolated myocytes were analysed using MATLAB algorithms that demarcate the cell outline from 405 nm-excited autofluorescence and define nuclear mask by thresholding Hoechst emission excited by the same wavelength. Only particles entirely within the field-of-view were analysed. The detected particles were quantified in terms of area and the long- and short-axis length. Criteria were set for myocyte area and long/short axis length ratio exclude dead (hyper-contracted) myocytes. Similarly, size and long/short axis ratio criteria were imposed for selecting nuclear areas. Fluorescence in the sequentially imaged 488 nm-excited channel was recorded and quantified in terms of (i) mean signal and its homogeneity in the cytoplasmic area, and (ii) mean signal in nuclear regions. Homogeneity was calculated using the formula below, where P is the grey-level co-occurrence matrix of N pixels normalized to have a sum of 1, with a mask applied to eliminate background:

$$\text{Homogeneity}\;=\overset{\text{N}}{\underset{\text{i}=1}{\Sigma }}\overset{\text{N}}{\underset{\text{j}=1}{\Sigma }}\frac{\text{P}(\text{i,j})}{1+|\text{i}-\text{j}|}$$

### 2.10 Permeabilization

Isolated myocytes with no prior EV pre-treatment were loaded with Hoechst dye (3.3 μM, 5 min), placed on a poly-L-lysine precoated superfusion chamber in Ca^2+^ free Tyrode containing (in mM): 135 NaCl, 20 HEPES, 4.5 KCl, 1 MgCl_2_, 11 glucose, 0.5 EGTA and pH of the solution adjusted 7.4 at 37 °C with NaOH. After stabilization, cells were super-fused with internal solution containing (in mM): 30 KCl, 90 potassium gluconate, 10 Hepes, 10 NaCl, 5 EGTA, 2 pyruvate, 2 glutamate, 2 malate, 5 MgATP, 0.25 ADP, 2.3 CaCl_2_, 1.092 MgCl_2_, 0.5 potassium phosphate and pH of this solution was adjusted 7.2 at 37 °C with KOH. These quantities were calculated to give 100 nM free Ca^2+^ and 1 mM free Mg^2+^. To permeabilize the surface membrane, cells were exposed to 0.005 % saponin-containing internal for 15 s. As a test of the protocol, some runs used cells loaded with calcein (4 μM, 5 min) to show complete release of intracellular dye.

### 2.11 Statistics

Statistical comparisons between treatment groups used one-way ANOVA with Dunnett’s T3 multiple comparison test. For analyses of data from isolated myocytes, hierarchical statistics were implemented to account for biological replicates and remove pseudo-replication error.

## 3 Results

**Fig. 1:**
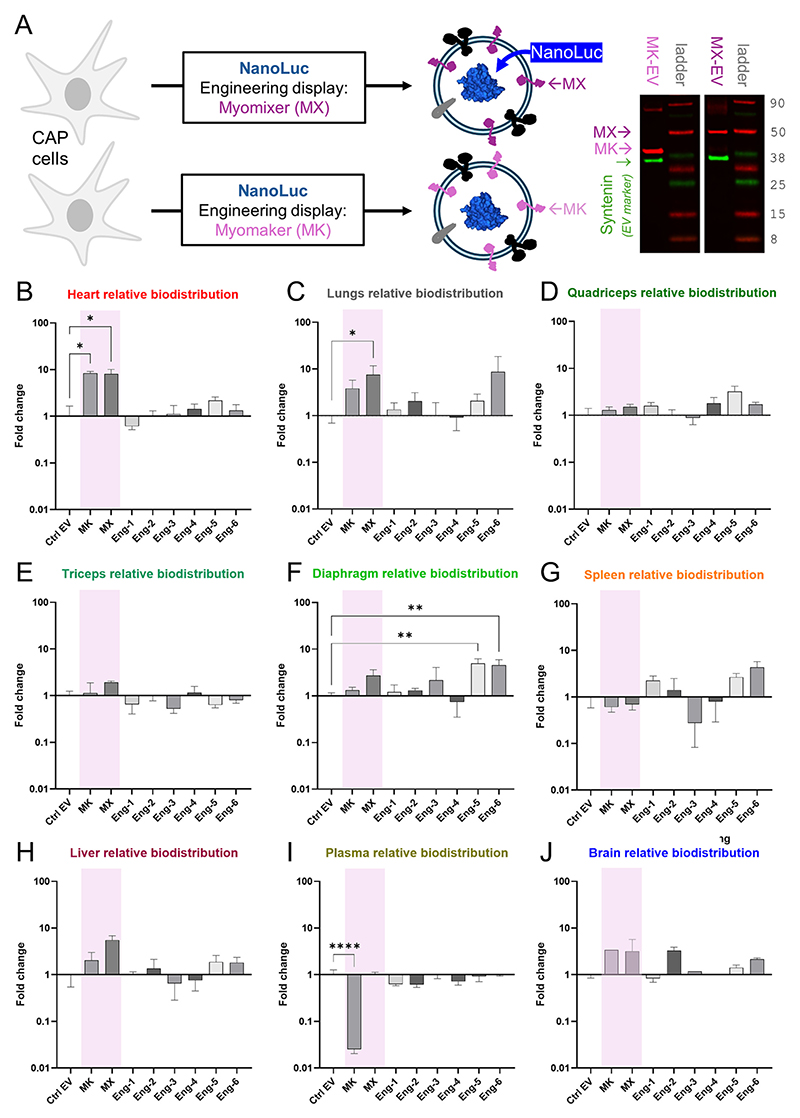
*In vivo* biodistribution of EV variants. (A) Generating EVs expressing NanoLuc (bioluminescence probe) and one of eight targeting proteins, including myomaker (MK) or myomixer (MX), verified by western blot. (B)-(J) Fold-change in luminescence measured in organs and plasma, relative to EVs without a specific targeting motif. Mean ± SEM, significance tested by one-way ANOVA followed by Dunnett’s T3 multiple comparison test *versus* first column (control). Organs obtained from 3 male and 3 female mice. ***P < 0.001, **P < 0.01, *P < 0.05.

Naturally secreted EVs have intrinsically poor myocardial targeting efficiency and undergo rapid clearance from the circulation *in vivo* when administered intravenously [30]. To seek improvements, we tested new EV designs delivered *via* Langendorff perfusion to mouse myocardium, and assessed uptake using readouts obtained from subsequently isolated myocytes, a process ensuring that measurements are purely from the intended target-cells and not stromal components such as capillaries or interstitial fluids. To test this discovery pipeline, we first sought reference EV designs for benchmarking subsequent improvements. First-pass experiments tested a range of EV constructs engineered to present various muscle-related proteins on their surface fused to LAMP2A, with NanoLuc at the C-terminus. These proteins included myomaker [31] and myomixer [32], as validated by western blot (Fig. 1A), and proteins identified previously as having muscle-binding affinity in [33] (Eng1), [34] (Eng2), [35] (Eng3), [36] (Eng4), [37] (Eng5) and [35] (Eng6). We reasoned that the EV design which produces significant cardiac enrichment is a suitable reference point for further improvements to targeting efficiency. To track biodistribution, EVs expressed the bioluminescent protein NanoLuc. Mice were injected with EVs and their organs harvested for measurements of light emission. Of all EVs tested, those engineered with myomaker or myomixer had 9-fold higher enrichment in the heart, compared to wild-type EV surfaces (Fig. 1B). Comparable enrichment was attained in the lungs for myomixer only (Fig. 1C). The cardiac result is likely a selective uptake because enrichment with myomixer/myomaker was not detected in other tissues, including skeletal muscles, the anticipated targets of these motifs (Fig. 1D-J). Leveraging the unexpected albeit modest cardiac-targeting specificity of myomaker and myomixer, we used these EVs as a reference for benchmarking surface modifications tested using a novel screening method.

**Fig. 2:**
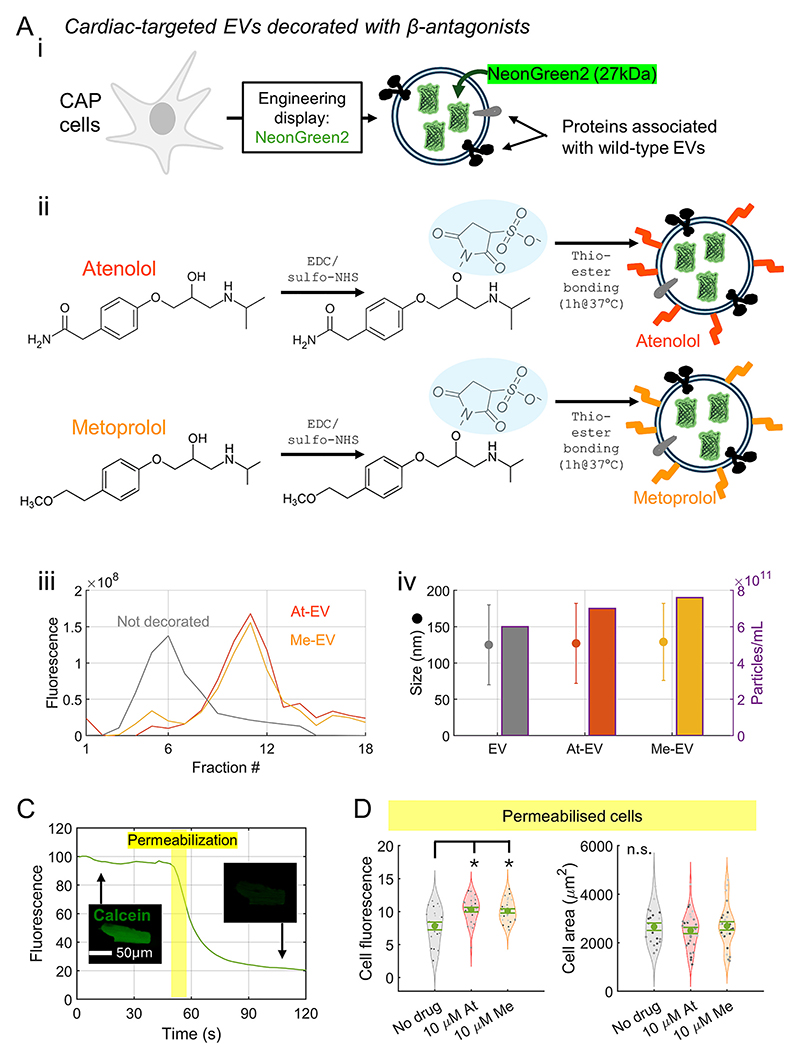
Producing and modifying extracellular vesicles (EVs). (A) (i) CAP cells transfected with NeonGreen2 produce EVs with fluorescent cargo. (ii) Using click-chemistry to decorate EVs with the sulfo-derivatives of either atenolol or metoprolol. (iii) Surface modification was confirmed by fluorescence measurements of fractionated EVs, showing a shift towards heavier fractions in decorated EVs. (iv) Decoration does not affect size (left axis) or yield (particle count) of EVs. Mean ± S. D. (B) EVs engineered to express NeonGreen2 and either myomaker or myomixer, proteins designed to target skeletal muscle but not explicitly cardiac muscle. (C) Saponin-permeabilization showing loss of calcein fluorescence in cardiac myocytes. (D) Permeabilization gives direct access from superfusate to mitochondria and other organelles. Myocytes obtained from 3 animals; each symbol represents one myocyte. A high concentration (10 μM) of atenolol (At) or metoprolol (Me) increased autofluorescence modestly. Cell area remained comparable to non-permeabilised myocytes, confirming the process did not cause hyper-contracture. Statistical testing by one-way ANOVA followed by pairwise comparisons. * indicates *P* < 0.05.

Our strategy for improving cardiac delivery considered methods to decorate the surface of EVs with small molecules known to bind to cardiomyocytes. To that end, we used the β_1_-antagonists atenolol and metoprolol, reasoning that these associate with beta-receptors but will not evoke a sympathomimetic response. Chemically modified EVs were benchmarked against EVs with an unmodified (wild-type) surface, characterised by CD63 expression [38], or EVs engineered with myomaker or myomixer. To track cargo delivery, EVs expressed Neon-Green2, a fluorescent protein emitting at 520 nm when excited by 488 nm laser light, instead of NanoLuc (Fig. 2Ai). NeonGreen2 fluorescence is >3 brighter, more photostable, and less pH-sensitive compared to green fluorescent protein [39]. Click chemistry was used to chemically bond β_1_-antagonists to EV-anchored proteins. Briefly, sulfo-derivatives of the drugs were produced by labelling with sulfo-NHS (N-hydroxysulfosuccinimide). This moiety enabled subsequent thio-ester bonding to free cysteine residues of proteins exposed at the EV surface (Figure 2Aii). This approach did not disrupt the aryl or amine groups that underpin selectivity properties for beta-receptors [40]. Incorporation of drugs was verified using fluorescence measurements on fractionated EV samples expressing NeonGreen2 (Fig. 2Aiii). Quality-control checks of EVs confirmed consistent particle size and concentration yield after modifications (Fig. 2Aiv), as well as evidence for strong fluorescence at settings also used for cardiomyocyte imaging experiments (Fig. S1).

A potential concern surrounding EVs decorated with β_1_-antagonists relates to a plausible inhibitory effect of certain β_1_-antagonists – including atenolol – on the respiratory chain [41]. This effect is not normally observed in intact myocytes because of low drug permeability across the surface membrane; however, cellular entry aboard EVs could expose cardiac mitochondria to the inhibitory moiety and introduce autofluorescence in the spectral range of NeonGreen2 [42]. To investigate the potential scope of this artefact, cardiomyocytes were saponin-permeabilised to expose mitochondria directly to β_1_-antagonists delivered by means of superfusion. In separate experiments, permeabilization was confirmed by demonstrating loss of fluorescence in calcein-loaded cardiomyocytes upon exposure to saponin (Fig. 2C). Permeabilized cells were superfused with internal solution containing ATP to prevent rigor, and mitochondrial substrates to facilitate respiration. Inclusion of atenolol or metoprolol at a high concentration (10 μM) produced a small but statistically significant effect on raising cardiomyocyte autofluorescence (Fig. 2D). This small effect-size fixes an upper limit for a potential artefact associated with respiratory inhibition that could arise from β_1_-antagonists released from internalized EVs.

**Fig. 3:**
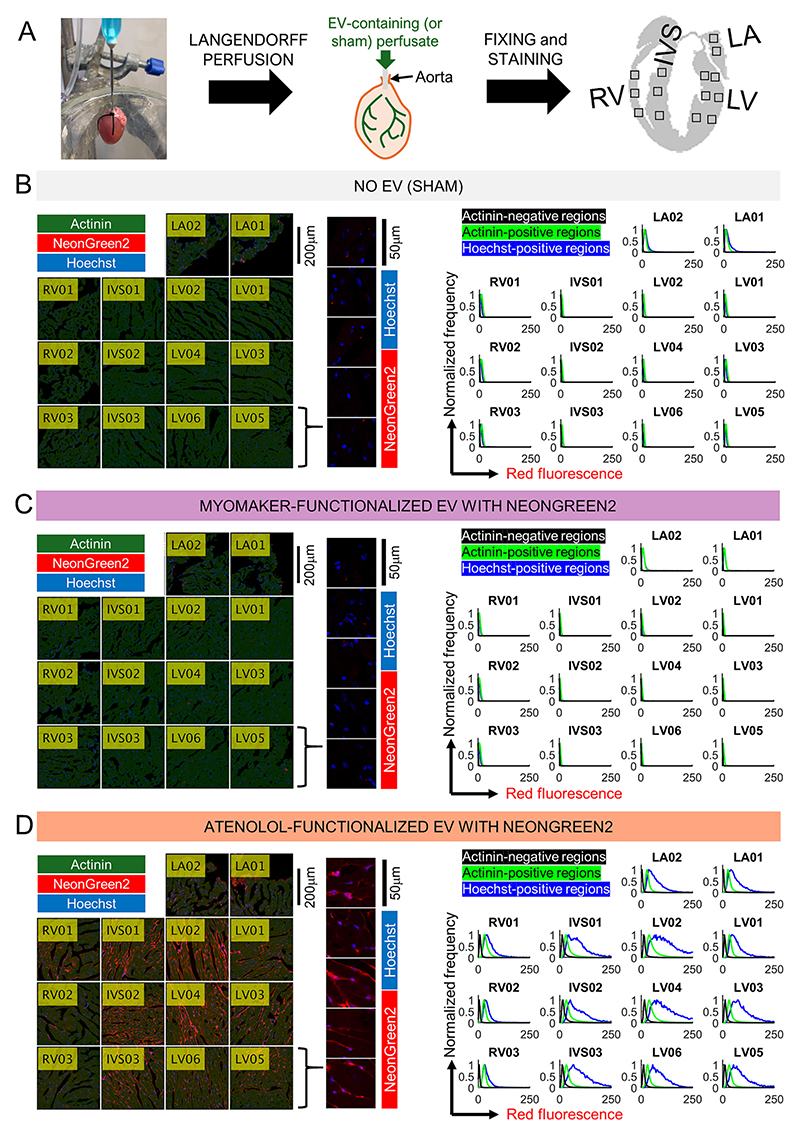
Immunofluorescence imaging of cardiac sections. (A) Protocol for Langendorff-perfusion with EVs, fixing and staining. Images were taken from different parts of the heart: left ventricle (LV), left atrium (LA), right ventricle (RV) and interventricular septum (IVS). (B) Immunofluorescence of cardiac sections after Langendorff-perfusion with EV-free solution (sham). Staining for α-actinin (green), NeonGreen2 (red) and Hoechst (blue). Green and blue channels were binarized. Inset shows images of LV-sections at higher magnification, without actinin channel. Histograms show quantification of NeonGreen2 immunofluorescence in actinin-negative regions (black), in actinin-positive/Hoechst-negative regions (green), and in nuclear regions (blue). Protocol and analysis repeated for heart after Langendorff-perfusion with (C) myomaker-presenting EVs (10^10^/ml; 1 h) or (D) atenolol-decorated EVs (10^10^/ml; 1 h). Note, immunoreactivity against NeonGreen2 in the atenolol group. Purple areas denote overlap between Hoechst fluorescence and NeonGreen2 immunopositivity. Exemplar images from two hearts per condition. (For interpretation of the references to colour in this figure legend, the reader is referred to the web version of this article.)

Our proposed discovery pipeline is complex and includes a Langendorff perfusion step followed by enzymic digestion and cell-based assays. To interrogate this protocol for flaws and limitations at an intermediate step, we assessed the uptake of β_1_-antagonist-decorated EVs expressing NeonGreen2 in fixed sections of adult mouse myocardium (Fig. 3A). The heart was excised immediately upon killing and Langendorff-perfused in recirculation-mode for 1 h at a concentration of 10^10^ particles/mL, followed by a 3-min washout period. As a control, sham experiments contained no EVs but perfusion parameters remained unchanged (Fig. 3B). For benchmarking, EVs containing myomaker were used (Fig. 3C). Next, hearts were fixed, sectioned, and stained for antibodies against α-actinin (green) and NeonGreen2 (red), as well as the dye Hoechst to indicate nuclei. Images were taken in 14 regions-of-interest spread across the left ventricle (LV), left atrium (LA), right ventricle (RV) and interventricular septum (IVS). In sham-perfusions or perfusions with myomaker-presenting EVs, myocardial NeonGreen2 immunoreactivity was not detectable in α-actinin-positive regions (Fig. 3B/C). In contrast, a heart perfused with atenolol-decorated EVs showed evidence for significant and widespread NeonGreen2 immunofluorescence, particularly in the left ventricle which is more accessible *via* the coronary vasculature from the aorta (Fig. 3D). Higher-magnification imaging of left ventricular sections revealed signal overlap between nuclear (Hoechst-positive) areas and NeonGreen2 immunopositivity, giving a purple appearance. Further analysis compared the statistical distribution of NeonGreen2 immunoreactivity gated by extracellular (α-actinin-negative), cytoplasmic (α-actinin-positive and Hoechst negative), or nuclear (Hoechst-positive) regions. Nuclear regions showed the strongest mean signal, indicating NeonGreen2 uptake into cells. Thus, these results demonstrate that β_1_-antagonist decorated EVs gain access to the myocardium and incorporate their cargo (Neon-Green2) into myocytes.

**Fig. 4:**
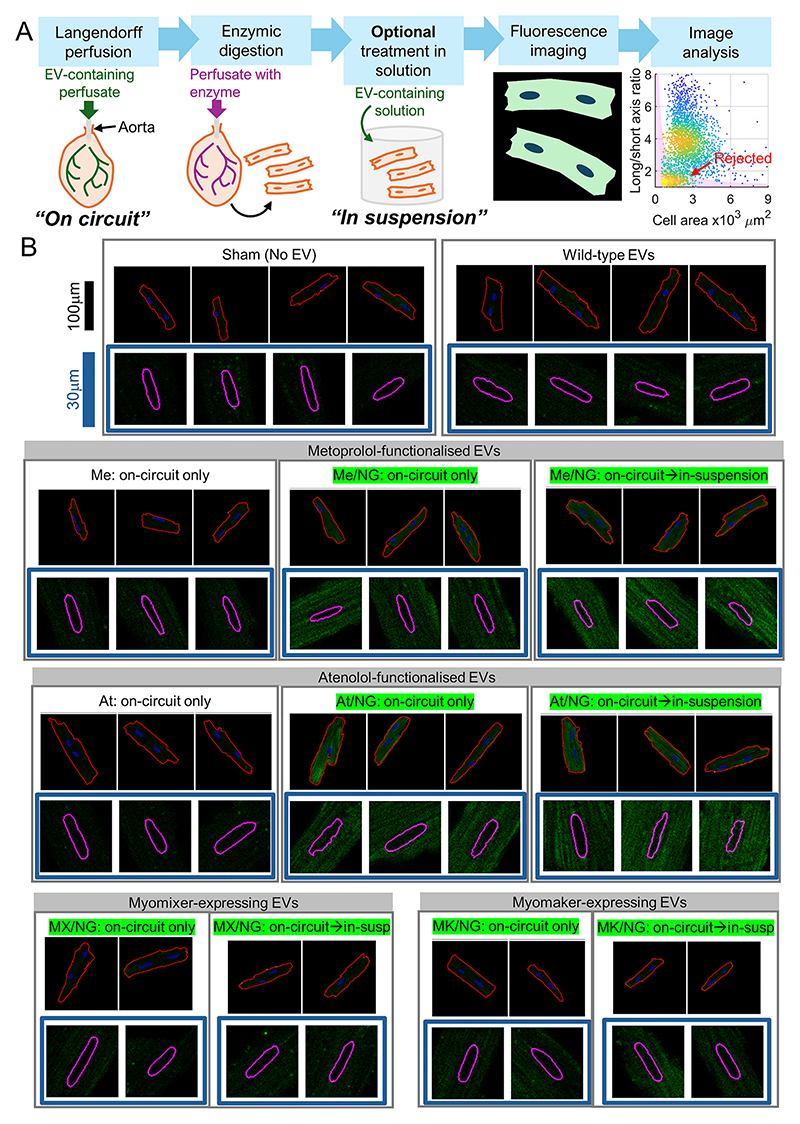
Langendorff delivery of EVs and subsequent cellular-level characterisation of cardiomyocyte uptake. (A) Protocol for Langendorff perfusion with EV-containing solution, followed by enzymic digestion to liberate cells, an optional period of loading cells in-suspension with EVs, and imaging of isolated cardiomyocytes. Image analysis separates rod-shaped myocytes based in criteria of area and long/short axis length ratio, from hypercontracted and dead cells (redshaded area). (B) Exemplar fluorescence images of cardiomyocytes (red outline) with nuclear mask (blue) and (blue frame) higher-magnification images of nuclear regions (nuclei shown in purple outline). Signal is detected at the emission peak of NeonGreen2. Conditions highlighted in green indicate that the EVs used for treatments expressed NeonGreen2 (NG). “On-circuit” indicates treatment with EVs during the period of Langendorff-perfusion; “In-suspension” indicates an additional treatment with the same EVs performed on myocyte suspensions, after enzymic digestion. (For interpretation of the references to colour in this figure legend, the reader is referred to the web version of this article.)

As a readout of EV efficacy, immunofluorescence in cardiac sections has limitations because it necessitates tissue fixing and precludes measurements on living cells. Additionally, it cannot accurately quantify uptake into cardiomyocytes on a cell-by-cell basis. To address these limitations, the Langendorff perfusion protocol was extended to include a digestion step whereby hearts, after EV treatment, were perfused with collagenase enzymes that liberate cardiomyocytes. The yield of cells can then be collected for imaging to assess NeonGreen2 fluorescence (excitation 488 nm, emission 520 nm) and any additional readouts that require living cells (Fig. 4A). Critically, this approach also removes any EVs that may remain in capillaries or interstitial fluids, *i.e*. a false-positive result that can compromise whole-organ or tissue lysate measurements. In the protocol, cardiomyocytes can be exposed to EVs in two instances: “on-circuit”, *i.e*., as EV-containing solution is re-circulated through Langendorff-perfused hearts prior to digestion and, optionally, “in-suspension”, *i.e*. in the solution that isolated myocytes are bathed after digestion.

For imaging, cells were loaded with Hoechst (excitation 405 nm, emission 460 nm) to identify nuclei (high signal) and to demarcate the cell outline from the weaker fluorescence signal associated with UV-evoked autofluorescence. Imaging was performed under superfusion to remove any EVs that would loosely attach to the surface, leaving only the cardiomyocyte response to EV integration. To ensure consistent acquisition and avoid bias, cells were focused to best capture nuclear regions on the Hoechst channel, rather than the NeonGreen2. Particles that met criteria for rod-shaped myocytes (area, long/short axis ratio) were analysed for cell-averaged NeonGreen2 fluorescence and its homogeneity. Within cells, Hoechst-positive regions that were within the expected range of nuclear size (>30 μm^2^) and long/short axis ratio (>3) provided a mask for analysing NeonGreen2 signal from the nucleus. Fig. 4B shows exemplar 520 nm fluorescence images from entire myocytes (indicated by red outline) and, inside blue frames, nuclear regions (indicated by purple outline). Images were taken of cardiomyocytes after treatment with wild-type (*i.e*. non-targeted) EVs, EVs decorated with metoprolol or atenolol, or EVs expressing either myomaker or myomixer. For some experiments, treatment was restricted to Langendorff perfusion (“on-circuit”) and in others, this was followed by treatment of isolated cells (“in-suspension”). On-circuit treatment allowed the myocardium to react with EVs in the context of capillary transit, which may place limits on the degree of exosome integration. Insuspension treatment permitted a much longer exposure that would investigate an upper limit for EV integration.

Control experiments used sham treatments (*i.e*., no EV dissolved) or EVs that lack NeonGreen2, *i.e*. are not expected to emit fluorescence after cellular internalization. A consistent increase in cellular fluorescence was noted in myocytes isolated from hearts that had been treated with EVs decorated with β_1_-antagonist and engineered to express NeonGreen2. This signal may represent fluorescence emitted directly from internalized NeonGreen2, or an autofluorescence response to the internalized cargo. Arguing in favor of the former source, treatment with β_1_-antagonist-decorated NeonGreen2-expressing EVs produced fluorescence within in-focus nuclear regions, which represents a distinct environment from the surround areas, notably devoid of mitochondria and lysosomes which are a major source of auto-fluorescence [43–46]. If at least some signal is attributable to NeonGreen2, a degree of recovery after localized photobleaching is expected form the limited diffusivity of the fluorescent protein. This was tested using a fluorescence recovery after photobleaching (FRAP) protocol performed on cardiomyocytes treated with atenolol-decorated, NeonGreen2-containing EVs. Bleaching fluorescence in a 10-by-10 μm region to 50 % of baseline signal resulted in a partial recovery, following a time constant of ~8 s, consistent with a small protein (Fig. S2). A large fraction of fluorescence did not recover, indicating an immobile emitter, such as anchored molecules, or molecules restricted to organellar structures.

**Fig. 5:**
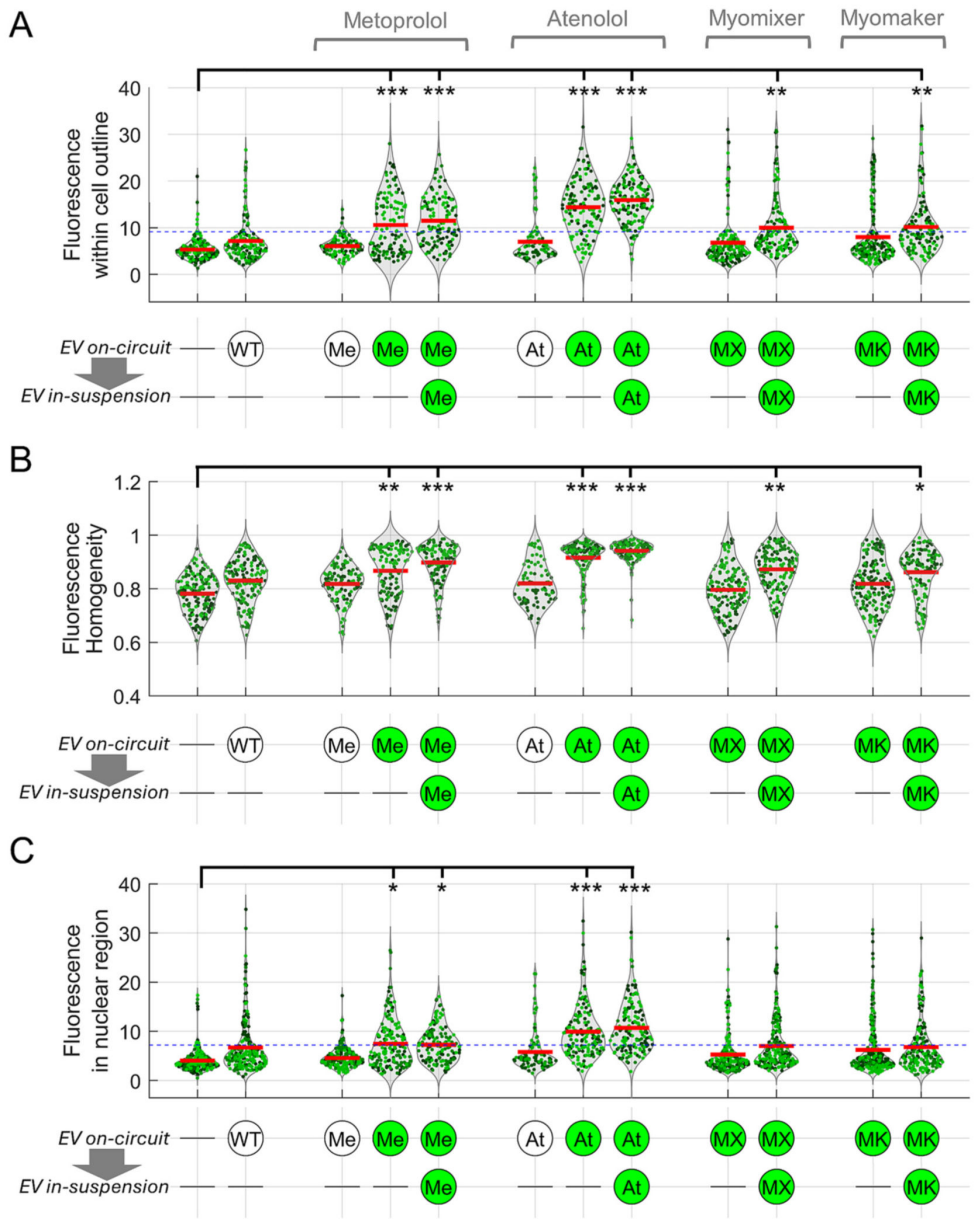
Quantification of cellular fluorescence responses to EV exposure. (A). Averaged fluorescence within myocytes that pass size and shape criteria for rod-shaped particles. Consistent imaging settings were used for accurate between-group comparisons. ANOVA testing by imaging session did not show significant batching effect. Red lines indicate mean. Blue dashed line is a threshold of positivity, determined from the highest cell-averaged value measured in sham treatment experiments (first column), after removing outliers from a Gaussian distribution. Below x-axis, circular icons indicate EV treatment during Langendorff perfusion (“on-circuit”) and subsequent treatment of cell suspensions (“in-suspension”), where applicable. Green-filled icons indicate EV was expressing NeonGreen2 fluorescent protein; empty icons are non-fluorescent. EVs tested included wild-type (WT), metoprolol-decorated (Me), atenolol decorated (At), expressing myomixer (MX) or myomaker (MK). (B) Analysis of cellular fluorescence in terms of homogeneity of signal. Fluorescence within the cell outline was analysed for the grey-level co-occurrence matrix to determine spatial relationships, from which homogeneity was derived as a metric of similarity between neighbouring pixels. A readout of 1 indicates uniformity; lower values indicate heterogeneity. (C) Average fluorescence signal from nuclear regions of myocytes, identified using the Hoechst-positive mask. Blue dashed line is a threshold of positivity, determined from the highest nucleus-averaged value measured in sham treatment experiments (first column), after removing outliers from a Gaussian distribution. Statistical testing with one-way ANOVA with hierarchical model to account for nesting of myocytes within a heart. *** = *P* < 0.001, ** = *P* < 0.01 and * = *P* < 0.05 relative to sham treatment. Myocytes obtained from 4 (sham) or 3 (all other) animals; each symbol represents one myocyte. (For interpretation of the references to colour in this figure legend, the reader is referred to the web version of this article.)

A quantification of imaging data is presented in Fig. 5, with each experiment performed on at leastthree hearts. The analyses are presented as cell-averaged fluorescence (Fig. 5A), its homogeneity based on neighbourhood relationships in pixel intensity (Fig. 5B), and nucleus-averaged fluorescence using Hoechst for masking (Fig. 5C). A threshold was calculated separately for cellular or nuclear fluorescence using images from sham experiments, after removing statistical outliers that fall outside a normal distribution. Treatment with wild-type EVs did not evoke a significant rise in fluorescence signal, or its cellular homogeneity. EVs decorated with β_1_-antagonists produced a robust increase in cellular signal that also became more homogeneous and produced a nuclear signal, but only if the EVs expressed NeonGreen2 as the putative source of fluorescence. Thus, EV internalization *per se* was deemed insufficient to produce a fluorescence response because signal was absent in β_1_-antagonist-decorated EVs lacking NeonGreen2. The degree of fluorescence increase was not increased further by additional in-suspension loading, which indicates that the bulk of the fluorescencerise took place during Langendorff perfusion. Compared to metoprolol-decorated EVs, atenolol-decorated EVs produced a more consistent response, described by a monomodal and near-Gaussian distribution of cellular and nuclear fluorescence. This difference may relate to the way that the drug molecules are incorporated by click-chemistry to the EV surface. Perfusion with myomixer- and myomaker-presenting EVs did not substantially increase cellular or nuclear fluorescence signals, unless EV treatment continued in the myocyte suspension. Nonetheless, the maximal effect with myomixer- and myomaker-presenting EVs was smaller than that attainable with beta-antagonist decorated EVs.

## 4 Discussion

Herein, we describe a method for assessing novel EV designs in terms of effectiveness in delivering cargo to cardiomyocytes. Our approach is based on Langendorff-perfused hearts *ex vivo*, a preparation that retains physiologically and anatomically relevant barriers, including coronary vasculature and stroma but allows full control over the EV dosage and exposure time. Langendorff perfusion can be followed immediately by enzymic digestion to obtain isolated myocytes for studying the cellular outcomes using assays available for *in vitro* studies. The outcomes of such a discovery pipeline eliminate extraneous variables, such as plasma half-life, that typically complicate the interpretation of animal studies, allowing a more robust assessment of true efficacy.

We tested the Langendorff-based method using EVs designed to bind β_1_-receptors on the premise that these are abundantly present in the heart [24]. Click chemistry was used to decorate EVs with β_1_-antagonists, and the results were benchmarked against wild-type EVs or EVs expressing motifs that do not have canonical cardiac targets. Although prior efforts have been made to functionalize EVs using click chemistry [47], this study is the first demonstration of click chemistry applied to functionalize EVs with β-antagonists. EVs decorated with atenolol or metoprolol were able to raise fluorescence signal inside isolated cardiomyocytes, including their nuclear regions, but only when expressing NeonGreen2 as their fluorescent cargo. This combination suggests that NeonGreen2 is either a source of cellular fluorescence or, ostensibly, a trigger of an autofluorescence response. Evidence supporting the former is the rise in fluorescence in nuclear regions of cells imaged confocally as well as immunofluorescence for NeonGreen2 epitopes detected in fixed cardiac sections using fluorescent secondary antibodies that have a redshifted emission compared to the fluorescent protein. Critically, wild-type EVs or EVs functionalised with canonical skeletal muscle proteins (myomaker, myomixer) did not produce a fluorescence response in isolated myocytes after a period of Langendorff perfusion. The improvement relative to myomaker and myomixer is significant, because these proteins conferred EVs a degree of cardiac targeting, as determined in first-pass *in vivo* experiments to identify reference designs for benchmarking improvements. The substantial increase in fluorescence with beta-antagonist decorated EVs indicates a significant improvement in design. Atenolol was found to be a more effective binder than metoprolol, which may relate to the manner in which the drug is integrated onto the EV surface and its degrees of freedom to bind effectively with the myocyte surface. Intriguingly, there was little added effect from a period of in-suspension treatment with EVs, indicating that Langendorff-loading may be particularly effective by juxtaposing cardiomyocytes close to circulating EVs. This scenario would increase the probability of collision, compared to the more stochastic process in suspension involving a much larger and dilute space. Critically, this latter limitation may expose a flaw of *in vitro* approaches to testing EV designs. Various factors affect how EVs are taken-up *via* endocytic pathways, including clathrin-dependent endocytosis, caveolin-mediated endocytosis, and micropinocytosis [48–52]. Beta-antagonist decoration may enhance these routes by creating favourable interactions with cell surface receptors [53]. We therefore hypothesise that the small molecule decoration is driving the increase in uptake by increasing the chance of EV interaction with cells displaying beta-1 adrenergic receptors. How that increased uptake affects the overall cell stress and, by extension, its autofluorescence is unknown in the exosome context. However, knowing the wide range of applications of NeonGreen2 as a fluorescent tag, there is little evidence to assume the cargo alone could evoke an autofluorescence stress response.

### 4.1 Limitations and future directions

The present study introduces a screening protocol for testing responses of the intact myocardium to perfusion with new EV designs. As proof-of-concept, EVs functionalized by click-chemistry with beta-blockers were verified to evoke a response specifically in cardiomyocytes. However, further experiments on mice are warranted to test if this EV design improves drug delivery to the heart *in vivo*. Beta-blocker functionalized EVs may not, for example, reach adequate bioavailability in a complete organism, or their interaction with myocytes may not deposit adequate levels of therapeutic cargo. Limiting factors not present in our method include liver clearance and dissipation across the wider circulation. Additionally, other types of beta-blockers or their derivatives may perform better than atenolol or metoprolol used herein, and should be tested. Whilst the method’s fluorescence readout is a useful indicator of the myocardium’s response to EVs, it cannot predict whether therapeutic cargo would produce a biological response. It remains unclear how the fluorescence response relates to the subcellular distribution of therapeutic payloads; for example, whether the drugs enter the cytoplasm or become trapped in endosomes. Further studies are required to compare the new EV design with existing products using specific drug payloads benchmarked against disease-relevant metrics. Nonetheless, our proposed screening protocol offers tangible benefits by selecting the most promising EV designs for animal studies. Moreover, the experimental control over variables attained with our protocol reduces experimental variability and improves power to resolve and troubleshoot causes of poor myocardial targeting ahead of *in vivo* testing. Thus, we propose the Langendorff-perfusion protocol as an intermediary step – nested between cellular *in vitro* studies and animal *in vivo* studies – that can accelerate discovery by removing spurious variables compromising the interpretation of EV efficacy measurements. By reducing experimental noise, our approach can expedite the translation of EV-based therapies.

## Data Availability

All data needed to evaluate the conclusions in the paper are present in the paper.

## Footnotes

Supplementary data to this article can be found online at https://doi.org/10.1016/j.yjmcc.2025.05.007.
